# Role of Calcium/Calcineurin Signalling in Regulating Intracellular Reactive Oxygen Species Homeostasis in *Saccharomyces cerevisiae*

**DOI:** 10.3390/genes12091311

**Published:** 2021-08-25

**Authors:** Guohui Li, Wenxuan Fu, Yu Deng, Yunying Zhao

**Affiliations:** 1 National Engineering Laboratory for Cereal Fermentation Technology (NELCF), School of Biotechnology, Jiangnan University, 1800 Lihu Road, Wuxi 214122, China; guohuili@jiangnan.edu.cn (G.L.); dengyu@jiangnan.edu.cn (Y.D.); 2 Jiangsu Provincial Research Center for Bioactive Product Processing Technology, Jiangnan University, 1800 Lihu Road, Wuxi 214122, China; 3 Key Laboratory of Industrial Biotechnology, Ministry of Education, Jiangnan University, 1800 Lihu Road, Wuxi 214122, China; 6190252001@stu.jiangnan.edu.cn

**Keywords:** *Saccharomyces cerevisiae*, reactive oxygen species (ROS), cell death, calcium/calcineurin signalling pathway, *AKR1*, *ERG3*

## Abstract

The calcium/calcineurin signalling pathway is required for cell survival under various environmental stresses. Using *Saccharomyces cerevisiae*, we explored the mechanism underlying calcium-regulated homeostasis of intracellular reactive oxygen species (ROS). We found that deletion of acyltransferase Akr1 and C-5 sterol desaturase Erg3 increased the intracellular ROS levels and cell death, and this could be inhibited by the addition of calcium. The hexose transporter Hxt1 and the amino acid permease Agp1 play crucial roles in maintaining intracellular ROS levels, and calcium induced the expression of the *HXT1* and *AGP1* genes. The cytosolic calcium concentration was decreased in both the *akr1Δ* and *erg3Δ* mutants relative to wild-type cells, potentially lowering basal expression of *HXT1* and *AGP1*. Moreover, the calcium/calcineurin signalling pathway also induced the expression of *AKR1* and *ERG3*, indicating that Akr1 and Erg3 might perform functions that help yeast cells to survive under high calcium concentrations. Our results provided mechanistic insight into how calcium regulated intracellular ROS levels in yeast.

## 1. Introduction

Calcium ions are intracellular signalling molecules that regulate many cellular processes in eukaryotic cells including programmed cell death, muscle contraction and cell proliferation [1]. The concentration of Ca^2+^ in organelles can be maintained in an optimal range via the calcium signalling pathway. For example, in *Saccharomyces cerevisiae* the cytoplasmic Ca^2+^ concentration is kept in the optimal range of 50–200 nM when it is lower than 1 μΜ or higher than 100 mM in the external environment [2,3]. Moreover, the calcium homeostasis system and signalling regulation pathway in yeast cells are conserved, and similar to those in mammalian cardiac muscle cells [4,5]. Therefore, the budding yeast *S. cerevisiae* provides powerful genetic and genomic tools for exploring calcium homeostasis and the signal transduction system in yeast cells, and the knowledge may be applicable to higher eukaryotes and useful for curing human diseases, such as pathological heart enlargement and heart failure [6,7].

Reactive oxygen species (ROS) play important roles in the regulation of cellular processes such as apoptosis and oxidative stress. Accumulated ROS inside cells cause oxidative damage to proteins, nucleic acids and other biological macromolecules [8]. Moreover, the accumulation of ROS plays important roles in cell death and necrosis in a variety of types of cells [9]. Importantly, oxidative damage and programmed cell death caused by ROS have been associated with serious human diseases such as heart disease, amyotrophic lateral sclerosis and other serious ailments [10]. In addition, ROS play crucial roles in cell aging and cancer development [11]. In aerobic respiration, electrons leak from the electron transport and generate ROS. Additionally, exposure to heavy metals, herbicides, ultraviolet radiation, chemicals, air pollution and other exogenous factors can also lead to ROS generation [12]. Many factors affect the transition from respiration to aerobic fermentation, including glucose transport, glycolytic enzymes and transcriptional factors that regulate the expression of genes involved in respiratory depression. In *S. cerevisiae*, glucose itself transmits respiratory inhibition signals through the Crabtree effect [13]. Glucose activates a series of signal transduction pathways involving G-protein-coupled receptors, hexokinases and transmembrane glucose receptors that work together to inhibit respiration and promote aerobic fermentation [14].

Mitochondria are the major site of energy metabolism in eukaryotic cells, and ATP is synthesised in these organelles through oxidative phosphorylation (ox-phos) [15]. Mitochondria are also the site of porphyrin and steroid hormones and lipid metabolism [16], and they play crucial roles in regulating intracellular Ca^2+^ homeostasis [17] and glucose sensing [18]. The main source of ROS is mitochondrial respiration, in which single electron reduction produces O_2_, superoxide anions and their derivatives [15]. Ca^2+^ is a ubiquitous signalling molecule in eukaryotic cells, and many pathological diseases are caused by Ca^2+^ imbalance in mitochondria [19]. Ca^2+^ overload in the mitochondrial matrix leads to the production of excessive ROS, the permeability transition pore of mitochondria is opened, and cytochrome C is released, which eventually leads to cell death [20,21]. When yeast cells are only stimulated by Ca^2+^, the increase in Ca^2+^ concentration in mitochondria promotes the production of more ATP in mitochondria by increasing respiratory chain activity, thus stimulating cell growth [15]. However, when the concentration of calcium ions exceeds a certain range, ROS levels in cells can increase, as occurs following stimulation by external pathogenic drugs, and in severe cases this can lead to cell death [22,23].

The absence of the *ERG3* gene, encoding a C-5 sterol desaturase, leads to the failure of sterol synthesis, resulting in a change in lipid composition in the cell membrane [24,25]. Furthermore, the permeability of the cell membrane is altered, more ROS enter the cell, and intracellular ROS levels increase [15]. *AKR1* encodes an integral membrane protein that is responsible for the palmitoylation of some proteins [26]. The HIP14/AKRL1 protein acyltransferase in mammals is a homolog of Akr1 that can modify several neuronal proteins [27,28]. In budding yeast, deletion of the *AKR1* gene prevents palmitoylation of Yck1 and Yck2, preventing them from being correctly located in the cytoplasmic membrane, resulting in increased intracellular respiration, production of excessive ROS, and ultimately cell death [29].

In our previous study, we identified mutants of *ERG3* and *AKR1* that were sensitive to high calcium stress [30]. Herein, we describe a new role for calcium in regulating intracellular ROS levels in *akr1∆* and *erg3∆* mutants. Deletion of *ERG3* or *AKR1* elevated intracellular ROS levels in yeast cells and calcium could inhibit this increase. The mechanism did not involve calcium-regulated restoration of membrane localisation of Yck1 and Yck2 but involved calcium-induced expression of *HXT1* and *AGP1*. In addition, calcium also induced the expression of *AKR1* and *ERG3*, and cytosolic calcium levels in *akr1∆* and *erg3∆* mutants were lower than in wild-type (WT) cells. We therefore speculate that, in WT cells, calcium induces *AKR1* and *ERG3* expression to protect cells against high calcium stress. When *AKR1* or *ERG3* is deleted, the cytosolic calcium concentration is reduced, which leads to decreased expression of *HXT1* and *AGP1*. Adding calcium upregulated *HXT1* and *AGP1* expression to reduce intracellular ROS levels to normal ranges.

## 2. Materials and Methods

### 2.1. Yeast Strains and Growth Media

The *S. cerevisiae* strains used in this study are listed in Table 1. YPD medium (2% *w*/*v* glucose, 2% *w*/*v* tryptone and 1% *w*/*v* yeast extract) was used to culture yeast cells under nonselective conditions. SC-URA or SC-HIS medium (0.17% *w*/*v* yeast nitrogen base, 2% *w*/*v* glucose, 0.5% ammonia sulphate) lacking uracil or histidine was used for maintenance of plasmids or selection, respectively. Solid media were prepared by adding 2% (*w*/*v*) agar to YPD or SC media when necessary. All yeast strains were cultured at 30 °C.

### 2.2. DNA Manipulations

To investigate the localisation of Yck1 and Yck2, the fusion proteins of GFP-Yck1, GFP-Yck2 were constructed in the WT BY4741, *akr1Δ*, and *erg3Δ* mutant strains, respectively. To achieve this, the HIS3MX6-P*_ADH1_*-GFP cassette was first amplified with primers YCK1-NGFP-F/YCK1-NGFP-R, YCK2-NGFP-F/YCK2-NGFP-R from plasmid pEASY-T1-HIS3MX6-P*_ADH1_*-GFP [32], respectively, and integrated into the WT BY4741, *akr1Δ*, or *erg3Δ* mutant strains. Correct integration was confirmed by PCR with primers GFP-check-F and YCK1-check-R or YCK2-check-R, respectively. To construct the Akr1-GFP and Erg3-GFP fusion proteins in the WT BY4741, the GFP-kanMX6 cassette was amplified with primers AKR1-CGFP-F/AKR1-CGFP-R or ERG3-CGFP-F/ERG3-CGFP-R from the plasmid pFA6a-GFP-kanMX6 [33], respectively. To overexpress *HXT1* or *AGP1* gene in *akr1Δ* and *erg3Δ* mutant strains, the HIS3MX6-P*_ADH1_* cassette was first amplified with primers HXT1-NF/HXT1-NR or AGP1-NF/AGP1-NR from HIS3MX6-P*_ADH1_*-GFP plasmid and then integrated into *akr1Δ* and *erg3Δ* mutants. The correct transformants were tested by PCR with primers HXT1-check-F/HXT1-check-R or AGP1-check-F/AGP1-check-R, respectively. All the transformants were screened in solid SD-HIS media. The correct integration was confirmed by PCR with primers AKR1-check-F/AKR1-check-R and ERG3-check-F ERG3-check-R, respectively. The primers were all listed in Table S1. Log phase cells expressing the indicated fusion proteins were first treated with or without 0.2 M CaCl_2_ for 60 min. All cells were visualised using a Nikon Eclipse 80i epifluorescence microscope.

To construct the *HXT1-lac*Z and *AGP1-lacZ* reporters, we first amplified the promoter of *HXT1* and *AGP1* with primer pairs HXT1-LF/HXT1-LR or AGP1-LF/AGP1-LR, respectively, and replaced it with the *PMR1* promoter in pRS316-*PMR1-lac*Z [34], yielding pRS316-*HXT1-lac*Z and pRS316-*AGP1-lac*Z.

### 2.3. ROS and Cell Death Assay

Yeast cells were first cultured overnight in YPD medium, then inoculated into fresh YPD medium to an initial OD_600_ of ~0.1. When cells had grown to the middle log phase, they were split into two aliquots, and cultured in the absence or presence of 0.2 M CaCl_2_ for an additional 2 h. Intracellular ROS levels and cell death were measured as previously described [35,36,37]. More than 500 cells were used each analysis, and data are presented as the mean ± standard deviation (SD) from three independent samples.

### 2.4. The β-Galactosidase Activity Assay

To measure P*_HXT1_*- and P*_AGP1_*-driven β-galactosidase activities, pRS316-*HXT1-lac*Z and pRS316-*AGP1-lac*Z plasmids were transformed into WT BY4741, *akr1Δ*, *erg3Δ* and *sod1Δ* mutant strains and were screened in solid SD-URA media. Transformants were first cultured overnight in SD-URA medium, inoculated into two aliquots of fresh YPD medium and grown to the middle log phase, then treated with or without 0.2 M CaCl_2_ for an additional 2 h. Yeast cells were harvested by centrifugation to extract total proteins, and *β*-galactosidase activity was measured as described previously [34]. Data are presented as the mean ± SD from six independent experiments.

### 2.5. RNA Extraction and qRT-PCR Analysis

For RNA preparation, the indicated yeast cells were first treated with or without 0.2 M CaCl_2_ for 2 h. Cells were then collected and total RNA was extracted by the hot phenol method [38]. First-strand cDNA was synthesised using a Primer Script RT Reagent Kit (CWBiotech, Beijing, China) according to the manufacturer’s instructions. The mRNA expression levels of *HXT1*, *AGP1*, *AKR1* and *ERG3* were estimated by PCR with the primer pairs listed in Table S1. The *ACT1* gene was used as an internal control. The results were analyzed through the −*ΔΔCt* method [39]. Each reaction was carried out in triplicate.

### 2.6. Cytosolic Calcium Concentration Measurements

To test the cytosolic calcium concentration, WT BY4741, *akr1Δ* and *erg3Δ* mutants were transformed with pEVP11-AEQ89 plasmid harbouring the aequorin protein [40]. Aequorin luminescence measurements were performed as described previously [32]. Luminescence was recorded every second by a Synergy H4 fluorescence reader (BioTek, Kocherwaldstr. 34, D-74177 Bad Friedrichshall, Germany) for 10 s before and 120 s after CaCl_2_ injection. Cytosolic free Ca^2+^ concentrations were calculated as described previously [41].

## 3. Results

### 3.1. Calcium Inhibits ROS and Cell Death in akr1Δ and erg3Δ Mutants

**Figure 1:**
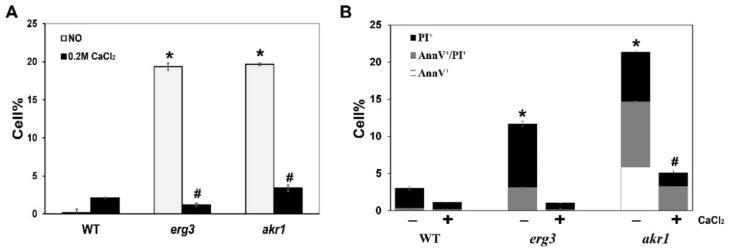
Calcium inhibits elevated ROS levels and cell death in *akr1∆* and *erg3∆* mutants. (**A**) Intracellular ROS levels in wild-type (WT) BY4741, *akr1∆* and *erg3∆* strains with or without calcium treatment. (**B**) Cell death in WT BY4741, *akr1∆* and *erg3∆* strains with or without calcium treatment. Results were analysed using paired-samples *t*-test function of SPSS 19.0. The significant difference of *p* < 0.01 is showed as “*” or “#” when cells were treated without or with calcium, respectively.

From a genome-wide screen, we previously identified 120 calcium-sensitive mutants [30]. To investigate how calcium regulates intracellular ROS levels, we investigated intracellular ROS levels in calcium-sensitive mutants. We found that cells lacking the C-5 sterol desaturase Erg3 and the integral membrane protein Akr1, which is responsible for palmitoylation of certain target proteins, exhibited elevated intracellular ROS levels compared with WT cells (Figure 1A). Interestingly, when calcium was added to the culture medium, the high intracellular ROS levels in *erg3∆* and *akr1∆* mutants could be inhibited (Figure 1A). Because intracellular ROS levels are closely related to cell death, we wondered whether mutants of *AKR1* and *ERG3* genes could also lead to greater cell death. We therefore assessed cell death in *erg3∆* and *akr1∆* mutants with or without calcium treatment. As shown in Figure 1B, cell death rates were higher in both *erg3∆* and *akr1∆* mutants than in WT cells, and calcium could also inhibit the elevated cell death in *erg3∆* and *akr1∆* mutants.

### 3.2. Akr1 and Erg3 Do Not Influence the Localisation of Yck1 and Yck2

**Figure 2:**
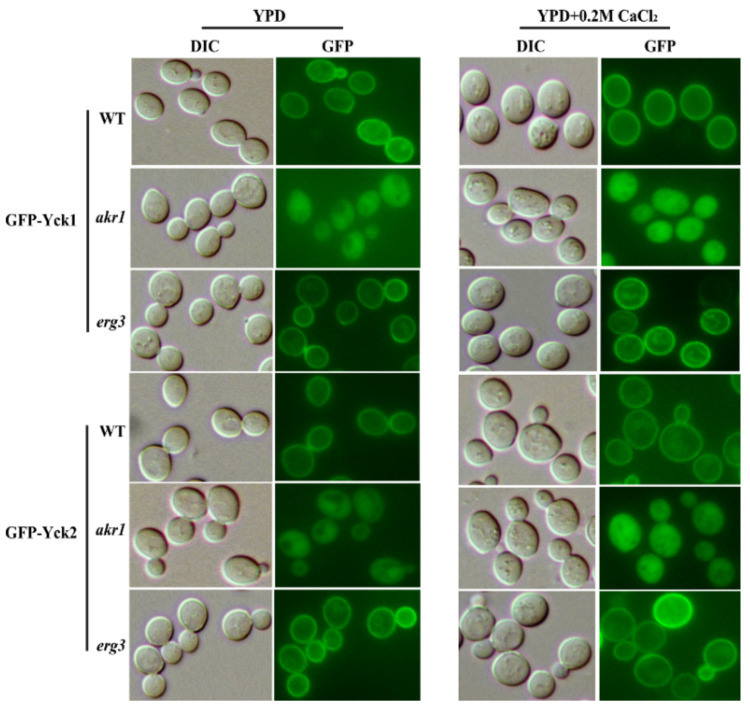
Mutants of *AKR1* and *ERG3* cannot recover cytosolic localisation of Yck1-GFP and Yck2-GFP. Wild-type BY4741, *akr1∆* and *erg3∆* cells expressing GFP-tagged Yck1 or Yck2 were grown to early log phase in YPD medium then were treated with 0.2 M CaCl_2_. Cells were visualised under a Nikon ECLIPSE 80i fluorescence microscope.

Yck1 and Yck2 are plasma membrane-associated casein kinase 1 (CK1) isoforms in yeast. Before Yck1 and Yck2 traffic to the plasma membrane, they must first be palmitoylated by Akr1 in the Golgi [42]. Yck1 and Yck2 are essential for inhibiting cellular respiration, and the deletion of these two genes can enhance respiration, causing cells to produce excessive oxygen free radicals, leading to cell death [43]. In *akr1∆* mutant cells, Yck1 and Yck2 cannot be palmitoylated and are therefore retained in the cytoplasm [44]. Here, we showed that calcium could inhibit elevated ROS levels and cell death in *erg3∆* and *akr1∆* mutants; hence, we evaluated whether the cytosolic localisation of Yck1 and Yck2 could be repaired by adding calcium. To achieve this, we tagged Yck1 and Yck2 with GFP at their N-termini in WT BY4741, *akr1Δ* and *erg3Δ* mutant strains to investigate their subcellular localisation. In WT BY4741 and *erg3Δ* mutant cells, Yck1-GFP and Yck1-GFP fusion proteins displayed a plasma membrane location with or without 0.2 M CaCl_2_ (Figure 2). In contrast, in *akr1Δ* mutant cells, Yck1-GFP and Yck1-GFP fusion proteins displayed a cytosolic localisation, and addition of calcium could not relocate these proteins to the plasma membrane. These results indicated that the inhibition of ROS generation and cell death in *akr1Δ* and *erg3Δ* mutant strains by calcium was not achieved by changing the cellular localisation of Yck1 and Yck2.

### 3.3. Calcium Induces the Expression of HXT1 and AGP1

The protein encoded by *HXT1* is a glucose-induced hexose transporter [45], and its expression is tightly regulated by the concentration of glucose in the culture medium [46]. *AGP1* is a gene encoding a low-affinity amino acid permease that is important for the uptake of amino acids as a nitrogen source in yeast cells [47,48]. The inhibition of the expression of these two genes leads to increased ROS levels in cells, leading to cell death. It has been reported that extracellular amino acids induced *AGP1* expression through the casein kinase isoforms Yck1 and Yck2 [49]. Moreover, the deletion of *AKR1* and superoxide dismutase 1 (*SOD1*) genes leads to decreased expression of *HXT1* and *AGP1* [50].

**Figure 3:**
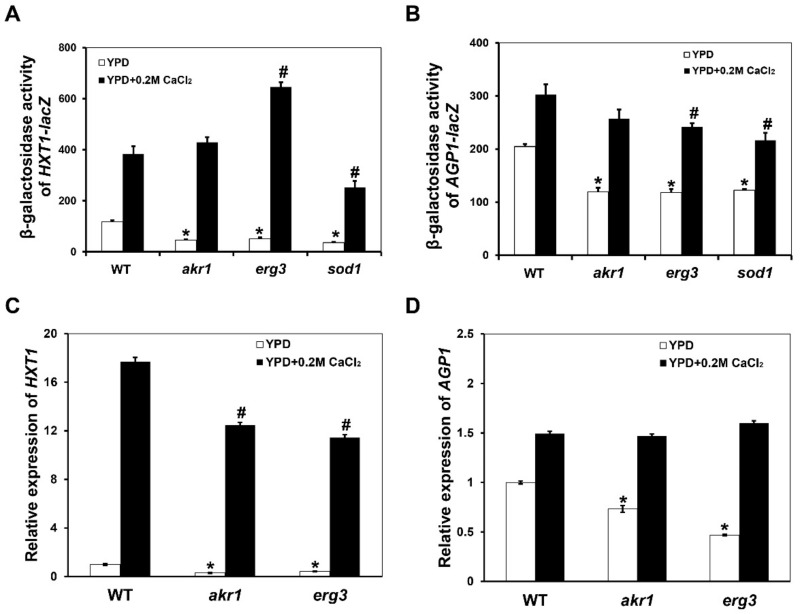
(**A**,**B**) *β*-galactosidase activity of *HXT1-lacZ* and *AGP1-lacZ* in WT, *akr1Δ*, *erg3Δ* and *crz1Δ* strains in response to 0.2 M CaCl_2_. Data are the means of six independent experiments. (**C**,**D**) Relative expression levels of *HXT1* and *AGP1* genes in response to 0.2 M CaCl_2_. WT, *akr1Δ* and *erg3Δ* strains were treated with 0.2 M CaCl_2_ for 2 h and expression of the indicated genes was measured by qRT-PCR. Values are averages of three independent assays for each strain. Results were analysed using paired-samples *t*-test function of SPSS 19.0. The significant difference of *p* < 0.01 is showed as “*” or “#” when cells were treated without or with calcium, respectively.

In order to investigate whether the inhibition of intracellular ROS levels and cell death in *erg3∆* and *akr1∆* mutants by calcium were related to the expression of *HXT1* and *AGP1* genes, we measured the expression levels of *HXT1* and *AGP1* in WT BY4741, a*kr1Δ*, *erg3Δ* and *sod1Δ* mutant strains. First, we constructed the *HXT1*-lacZ and *AGP1*-lacZ reporter plasmids and measured their expression levels through *β*-galactosidase activity assays. As shown in Figure 3A,B, the expression of both *HXT1*-lacZ and *AGP1*-lacZ was significantly reduced in *akr1Δ*, *erg3Δ* and *sod1Δ* mutants compared with WT cells in YPD medium. When yeast cells were treated with 0.2 M CaCl_2_, the *β*-galactosidase activities of *HXT1*-lacZ and *AGP1*-lacZ reporters were stimulated in WT BY4741, *akr1Δ*, *erg3Δ* and *sod1Δ* mutant strains. Specifically, the expression level of *HXT1-lacZ* in *akr1Δ* and *erg3Δ* mutants were both higher than that of the WT BY4741 cells after calcium treatment. Although the expression level of *AGP1-lacZ* was slightly lower in *akr1Δ* and *erg3Δ* mutants than that of the WT BY4741 cells after induction by calcium (0.85- and 0.8-fold of WT, respectively), this difference is relatively smaller than when there was no calcium treatment (both 0.59 fold of WT in *akr1Δ* and *erg3Δ* mutants).

To verify the induction of *HXT1* and *AGP1* by calcium, we measured the relative expression levels of *HXT1* and *AGP1* genes by the qRT-PCR method. Expression levels of *HXT1* and *AGP1* were significantly up-regulated after treatment with 0.2 M CaCl_2_ in WT BY4741, *akr1Δ* and *erg3Δ* mutant cells (Figure 3C,D), consistent with the results of expression of the *AGP1*-lacZ and *HXT1*-lacZ reporters. These results indicate that elevated intracellular ROS levels and cell death in *akr1Δ* and *erg3Δ* mutant cells might be caused by decreased expression of *HXT1* and *AGP1*, and calcium could inhibit these phenomena by inducing the expression of *HXT1* and *AGP1*.

**Figure 4:**
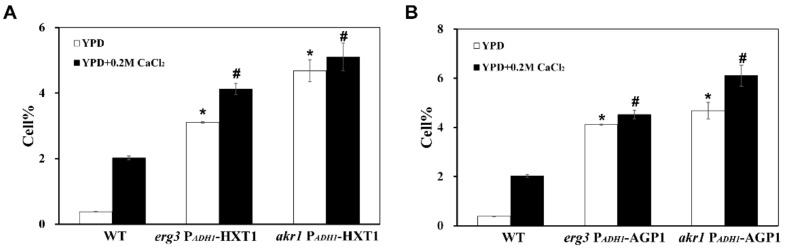
Overexpression of *HXT1* or *AGP1* could inhibit the increased intracellular ROS levels in *akr1∆* and *erg3∆* mutants. (**A**) Intracellular ROS levels in wild-type (WT) BY4741, and *akr1∆* and *erg3∆* mutants overexpressing *HXT1*. (**B**) Intracellular ROS levels in wild-type (WT) BY4741, and *akr1∆* and *erg3∆* mutants overexpressing *AGP1*. Results were analysed using paired-samples *t*-test function of SPSS 19.0. The significant difference of *p* < 0.01 is showed as “*” or “#” when cells were treated with or without calcium.

To investigate whether the high ROS levels are due to the low *HXT1* or *AGP1* expression, *HXT1* or *AGP1* were overexpressed in *akr1Δ* and *erg3Δ* mutants, respectively. As shown in Figure 4, the overexpression of both *HXT1* and *AGP1* could significantly reduce the elevated intracellular ROS levels in *akr1Δ* and *erg3Δ* mutants, although the ROS levels were still higher than that of WT cells. These results indicated that the expression levels of both *HXT1* and *AGP1* were closely related to the intracellular ROS levels of *akr1Δ* and *erg3Δ* mutants.

### 3.4. Calcium Positively Regulates AKR1 and ERG3 Expression

**Figure 5:**
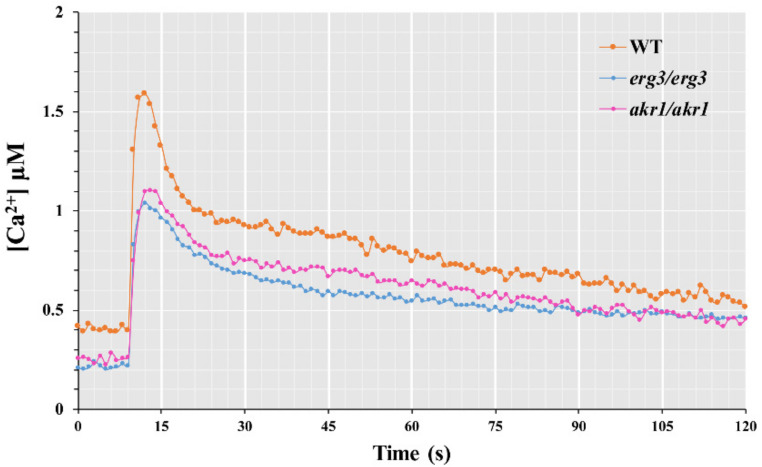
Cytosolic Ca^2+^ levels are decreased in *akr1Δ* and *erg3Δ* strains. WT, *akr1Δ* and *erg3Δ* strains harbouring the pEVP11-AEQ plasmid were used to monitor cytosolic Ca^2+^ concentrations. Data are the means of three independent experiments.

Since *akr1Δ* and *erg3Δ* mutants are sensitive to calcium [30], and because calcium can inhibit elevated intracellular ROS levels and cell death caused by the deletion of *AKR1* and *ERG3*, we wondered why *akr1Δ* and *erg3Δ* mutants are sensitive to calcium. One reason might be that deletion of *AKR1* or *ERG3* can cause increased cytosolic calcium levels that lead to *akr1Δ* and *erg3Δ* mutants being sensitive to calcium responses. The other reason might be that calcium can activate *AKR1* and *ERG3* to perform functions that help mutant cells to survive in response to high concentrations of calcium. To test these hypotheses, we first investigated cytosolic calcium levels in WT BY4741, *akr1Δ* and *erg3Δ* mutant strains using the pEVP11-AEQ89 plasmid [40]. As shown in Figure 5, the cytosolic calcium concentrations in both *akr1*Δ and *erg3*Δ mutants were lower than in the WT BY4741 strain. This result might explain why expression of *HXT1* and *AGP1* was lower than in WT BY4741 without calcium treatment.

**Figure 6:**
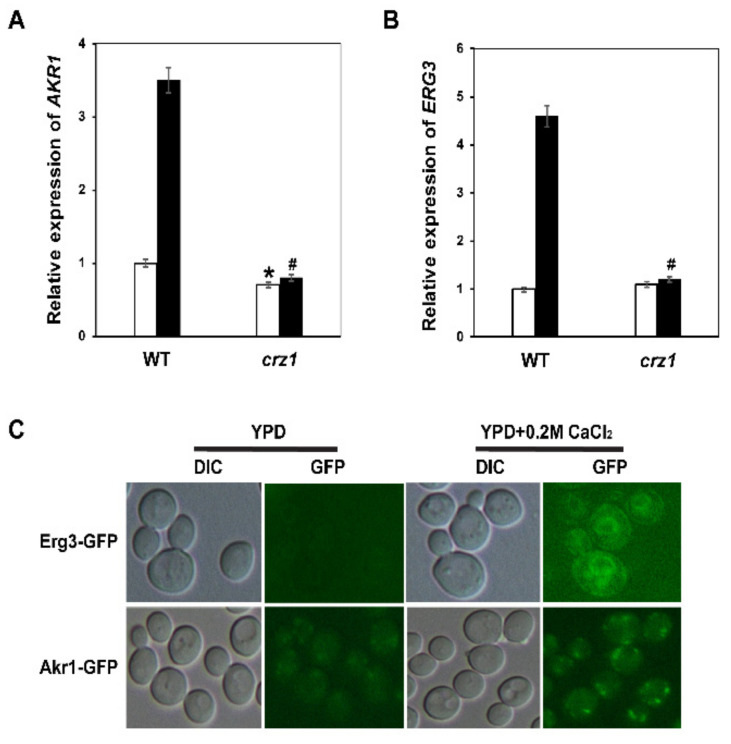
Expression levels of *AKR1* and *ERG3* are regulated by the calcium/calcineurin pathway. (**A**) Relative expression levels of the *AKR1* gene in response to 0.2 M CaCl_2_. (**B**) Relative expression levels of the *ERG3* gene in response to 0.2 M CaCl_2_. WT and *crz1Δ* strains were treated with 0.2 M CaCl_2_ for 2 h and expression of the indicated genes was measured by qRT-PCR. Values are averages of three independent assays for each strain. (**C**) Subcellular localisation of Erg3-GFP and Akr1-GFP proteins in response to 0.2 M CaCl_2_. Wildtype BY4741 cells expressing Erg3-GFP or Akr1-GFP were grown to early log phase in YPD medium then treated with 0.2 M CaCl_2_. Cells were visualised under a Nikon ECLIPSE 80i fluorescence microscope. Results were analysed using paired-samples *t*-test function of SPSS 19.0. The significant difference of *p* < 0.01 is showed as “*” or “#” when cells were treated without or with calcium, respectively.

Because the reduced cytosolic calcium concentrations in *akr1Δ* and *erg3Δ* mutants are not the cause of calcium sensitivity, we next tested whether expression of *AKR1* and *ERG3* could be induced by calcium. The expression levels of *ERG3* and *AKR1* genes in BY4741 and *crz1Δ* strains were not significantly different without calcium treatment. Expression levels of *ERG3* and *AKR1* genes were significantly induced by calcium, but there was no significant difference in expression of *ERG3* and *AKR1* genes in *crz1Δ* after calcium treatment (Figure 6A,B).

We also analysed the subcellular localisation of Akr1-GFP and Egr3-GFP in WT BY4741 cells with or without calcium treatment. As shown in Figure 6C, expression of Akr1-GFP and Egr3-GFP was very low and their fluorescent location signals were very weak. After adding 0.2 M CaCl2, the Akr1-GFP fusion protein could be clearly observed in the Golgi apparatus, while the Erg3-GFP fusion protein was localised in the endoplasmic reticulum as previously reported [51]. These results indicated that calcium regulated the expression of *ERG3* and *AKR1* genes through the transcriptional factor Crz1.

## 4. Discussion

In this study, we found that mutants of C-5 sterol desaturase Erg3 and palmitoyl transferase Akr1 involved in protein palmitoylation could cause elevated intracellular ROS levels and cell death. Furthermore, the addition of calcium reversed these phenomena in the *akr1Δ* and *erg3Δ* mutant strains. The deletion of *ERG3* and *AKR1* led to reduced cytosolic calcium content and a consequent decrease in expression of the hexose transporter *HXT1* and the amino acid permease *AGP1*. The addition of calcium induced expression of *HXT1* and *AGP1* to levels observed in WT cells, which helped to protect *akr1Δ* and *erg3Δ* mutant cells against elevated intracellular ROS and cell death. In addition, calcium induced the expression of *ERG3* and *AKR1* through the transcriptional factor Crz1, implying that Akr1 and Erg3 performed functions that helped yeast cells to survive in response to high concentrations of calcium.

Expression of the yeast low-affinity glucose transporter Hxt1 is regulated by glucose availability: it is activated when there is glucose in the culture medium and inhibited when glucose is scare [52]. In budding yeast, three signalling pathways (Rgt2/Snf3, AMPK and cAMP-PKA) are employed to sense glucose in the culture medium. When glucose was added to glucose-starved or glucose-derepressed cells, it has been reported that Gpr1/Gpa2 and Ras complex which can activate cAMP are needed for this process [53]. In the absence of glucose, the three pathways are inactivated by the Rgt1 repressor, which represses *HXT1* expression by recruiting the corepressor complex Ssn6-Tup1 and the corepressor Mth1 to the *HXT* promoters [54,55,56]. Therefore, multiple signalling pathways have been reported to regulate *HXT1* expression. First, the TOR kinase pathway and the PP2A protein phosphatase complex might be involved in glucose-induced *HXT1* expression [57,58]. Secondly, PKA plays important roles in maintaining the stability of Hxt1 by regulating its turnover in response to glucose starvation [46]. In addition to transcriptional regulation, Hxt1 and Agp1 can also be regulated post-translationally. For instance, Hxt1 and Agp1 can be internalised and degraded in the vacuole [59,60]. Grr1, one of the F-box components of the SKp1/cullin/F-box (SCF, one class of E3 enzymes) protein is involved in induction of *HXT1* and *AGP1* expression by interacting with Rgt1 in response to glucose and exogenous amino acids, respectively [61,62]. Herein, we demonstrated that calcium signalling was also involved in the expression of *HXT1* and *AGP1*, indicating that calcium signalling was also involved in the regulation of glucose-related respiration.

In yeast cells, an important function of the calcium/calcineurin-dependent signalling pathway is to regulate the expression of genes by activating the transcription factors Crz1/TCN1/Hal8 [63,64,65]. The zinc finger motif of the activated Crz1 then specifically binds to the calcineurin-dependent response element (CDRE; conserved sequence 5′-GNGGC(G/T)CA-3′) in the promoter, a 24 bp DNA sequence that is essential for the calcium/calcineurin-dependent induction of gene expression [63]. Calcium/calcineurin and Crz1 regulate various genes, including those encoding membrane-binding proteins, plasma membrane proteins and protein components of the cell wall [66]. Genes encoding proteins participating in vesicle transport, lipid/sterol synthesis and protein degradation, cell wall integrity and ion and small molecule transport are also regulated by the calcium/calcineurin signalling pathway. The calcium/calcineurin pathway also induces the expression of genes involved in key components in this and other signalling pathways, which provides various feedback mechanisms regulating this pathway and crosstalk with other signalling pathways [66]. In the present study, we demonstrated that calcium regulates the expression of *HXT1*, *AGP1*, *AKR1* and *ERG3*. Analysis of the promoter sequences of these four genes revealed that all contain predicted CDREs in their promoter regions. Therefore, we concluded that calcium-regulated expression of these four genes mainly acted through the transcription factor Crz1 that binds CDRE motifs in their promoters. In addition, since mitochondrial respiration is a main source of ROS [15], and since mitochondria also play crucial roles in regulating intracellular calcium homeostasis [17] and glucose sensing [18], thus, the link between calcium and ROS homeostasis as well as mitochondrial respiration needs to be further studied.

## 5. Conclusions

In conclusion, our results unveiled a new role for calcium/calcineurin signalling in regulating intracellular ROS homeostasis. By inducing the expression of *HXT1* and *AGP1*, calcium inhibited the elevated intracellular ROS levels and cell death in *akr1∆* and *erg3∆* mutants. Our studies will help elucidate the mechanisms by which the calcium/calcineurin pathway regulates intracellular ROS levels and cell death in budding yeast.

## Figures and Tables

**Table 1:** Strains used in this study.

Name	Relevant Genotype	Source/Reference
BY4741	MATa *his3Δ1 leu2Δ0 met15Δ0 ura3Δ0*	[31]
*akr1Δ*	BY4741 *akr1::kanMX4*	[31]
*erg3Δ*	BY4741 *erg3::kanMX4*	[31]
*sod1Δ*	BY4741 *sod1::kanMX4*	[31]
*crz1Δ*	BY4741 *crz1::kanMX4*	[31]
BY4741 GFP-YCK1	BY4741 *HIS3MX6- GFP-YCK1*	This study
*akr1Δ* GFP-YCK1	BY4741 *akr1::kanMX4 HIS3MX6-GFP-YCK1*	This study
*erg3Δ* GFP-YCK1	BY4741 *erg3::kanMX4 HIS3MX6-GFP-YCK1*	This study
BY4741 GFP-YCK2	BY4741 *HIS3MX6-GFP-YCK2*	This study
*akr1Δ* GFP-YCK2	BY4741 *akr1::kanMX4 HIS3MX6-GFP-YCK2*	This study
*erg3Δ* GFP-YCK2	BY4741 *erg3::kanMX4 HIS3MX6-GFP-YCK2*	This study

## Data Availability

The data used to support the findings of this study are included within the article and the Supplementary Materials.

## Supplementary Materials

The following are available online at https://www.mdpi.com/article/10.3390/genes12091311/s1, Table S1: Primers used in this study.
