# Supplementary material for: Role of Calcium/Calcineurin Signalling in Regulating Intracellular Reactive Oxygen Species Homeostasis in Saccharomyces cerevisiae
Source: Genes (Basel). 2021 Aug 25;12(9):1311. doi: 10.3390/genes12091311 (PMC8466207; doi:10.3390/genes12091311)
Supplement: Supplementary file 1 [file genes-12-01311-s001.zip › genes-1298088-supplementary.pdf]

Table S1. Primers used in this study

Name	Sequence (5′-3′)
YCK1-NGFP-F	TTTGATTTCTGCTTGCTTCTTACAAACAACAAACGCAAACCGTTCATTGA GAATTCGAGCTCGTTTAAAC
YCK1-NGFP-R	TTGGTGAGGTTGTTAACTGCTAGAGTGGTACTTGCTATGGGCATGGACA TTTTGTATAGTTCATCCATGC
YCK2-NGFP-F	ATTGTTTTTCTCTTTCGAGATTTAAGAACGTGGTTGTGTTTCGTGTTTCT GAATTCGAGCTCGTTTAAAC
YCK2-NGFP-R	ACAGCTAAACCAGAGTTCGTTGCTGTCAAAGGACTTTGCACTTGAGACATT TTGTATAGTTCATCCATGC
GFP-check-F	TGGAGAGGGTGAAGGTGATG
YCK1-check-R	CCGGAATTCCTGGTGGTGGAGTTGTTTG
YCK2-check-R	CCGGAATTCAGAGCTATTGCCAGTGTTGTG
AKR1-CGFR-F	AGCTTTACAAATTGCCAAATAAGGATGTTGAACAAGGAAATGATATGGTA CGGATCCCCGGGTTAATTAA
AKR1-CGFR-R	CTTCCAGTAATTTCCCAAAACAAACATATGTATATTATATATATATGTGC GAATTCGAGCTCGTTTAAAC
ERG3-CGFR-F	ATGATAATGATAGAATCTATGAAAACGACCCAAATACCAAGAAGAACAAC CGGATCCCCGGGTTAATTAA
ERG3-CGFR-R	AGCGCATATTGCACTAACGTGAGGTGTACATCATAATGATATGCGTATCT GAATTCGAGCTCGTTTAAAC
AKR1-check-F	ATCAAGGATAGCACCGGAC
AKR1-check-R	ATGATCGTTTTCTCTTACTCGC
ERG3-check-F	TTCTTACCGTAGACCAGATGAC
ERG3-check-R	TGCTTATAGAGTAGTGGCGGC
HXT1-NF	CGTTTTTAATCATTTGAATTAGTATATTGAAATTATATATAAAGGCAACA GAATTCGAGCTCGTTTAAAC
HXT1-NR	TTGGATGAATTGGATTTCTGAGGAGATATTAGATCGGGAGTTGAATTCAT TGTATATGAGATAGTTGATTGTATGC
AGP1-NF	TTTTGTTATTTTCCTCGTAATACTCATTTGTTTTACATACATATATAAGT GAATTCGAGCTCGTTTAAAC
AGP1-NR	GAGCTATTTTTCAAGTCTTTCAGTTCGTATAGAGACTTCGACGACGACAT TGTATATGAGATAGTTGATTGTATGC
HXT1-check-F	AACTTCAATTCATATCGACCGAC
HXT1-check-R	TGAACGACCAGATTCCAATTC
AGP1-check-F	ACGAAGGTGTGCATTCTCCC
AGP1-check-R	GGATGGACGGTCATTGGAG
HXT1-LF	CCCTCACTAAAGGGAACAAAAGCTGGGTACCACTTCAATTCATATCGACCGAC
HXT1-LR	ACGACGGGATCGCAAGCTGGGAATTCTGAACGACCAGATTCCAATTC
AGP1-LF	CCCTCACTAAAGGGAACAAAAGCTGGGTACCCGAAGGTGTGCATTCTCCC
AGP1-LR	ACGACGGGATCGCAAGCTGGGAATTCGGATGGACGGTCATTGGAG
HXT1-RT-F	TTGGTTGTGCCATTGGTGGTATCGTC
HXT1-RT-R	TGGAACTCTCCATTGGACAGAGTTTGAG

AGP1-RT-F	ATGTTCTGATGATTGCGTTGGGTACGG
AGP1-RT-R	ACCAATTCCAGAGGACACACACACAGCC
AKR1-RT-F	TAACGACGGTGACTCTACGG
AKR1-RT-R	TATCTGCGATCTTTATGCTGG
ERG3-RT-F	ACACCTTTCGCATCTCATTC
ERG3-RT-R	TTATCATCACCTTCGACCTCC
ACT1-RT-F	CTGAATTAACAATGGATTCTG
ACT1-RT-R	TTCCATATCGTCCCAGTTGG
